# The Impact of Rate Formulations on Stochastic Molecular Motor Dynamics

**DOI:** 10.1038/s41598-019-54344-2

**Published:** 2019-12-05

**Authors:** R. Blackwell, D. Jung, M. Bukenberger, A.-S. Smith

**Affiliations:** 1 PULS group, Physics Department and Interdisciplinary Center for Nanostructured Films, Friedrich-Alexander University Erlangen-Nürnberg, Cauerstrasse 3, 91058 Erlangen, Germany; 2 Group for Computational Life Sciences, Division of Physical Chemistry, Insitut Rūder Bošković, Bijenička cesta 54, 10000 Zagreb, Croatia

**Keywords:** Computational science, Biological physics

## Abstract

Cells are complex structures which require considerable amounts of organization via transport of large intracellular cargo. While passive diffusion is often sufficiently fast for the transport of smaller cargo, active transport is necessary to organize large structures on the short timescales necessary for biological function. The main mechanism of this transport is by cargo attachment to motors which walk in a directed fashion along intracellular filaments. There are a number of models which seek to describe the motion of motors with attached cargo, from detailed microscopic to coarse phenomenological descriptions. We focus on the intermediate-detailed discrete stochastic hopping models, and explore how cargo transport changes depending on the number of motors, motor interaction, system constraints and rate formulations, which are derived from common thermodynamic assumptions. We find that, despite obeying the same detailed balance constraint, the choice of rate formulation considerably affects the characteristics of the overall motion of the system, with one rate formulation exhibiting novel behavior of loaded motor groups moving faster than a single unloaded motor.

## Introduction

Motor proteins are ubiquitous agents of active motion in biological systems, powering such diverse processes as the flagellar propulsion of sperm cells^1^, the contraction of muscle fibres via myosin^2^ or the transport of neurotransmitters by kinesin^3^. While studied for a multitude of reasons, two stand out for why they are of considerable research interest. First, since motor proteins play an important role in such a wide variety of biological processes, it is not surprising that disturbances in the function of these molecular motors can cause an equally wide variety of medical problems, such as primary ciliary dyskinesia^4^, Bardet-Biedel syndrome^5^, Charcot-Marie-Tooth disease^6^, motor neuron disease^7^, Alzheimer’s^8^, ALS^9^, and others. Second, they serve as an inspirational blueprint in the nascent engineering and development of synthetic molecular machines^10–12^. Such synthetic motors would not only be useful due to their microscopic size per se, but would also promise an energy efficiency noticeably larger than their macroscopic counterparts. For example, the ubiquitous kinesin motor effectively uses approximately 60% of the chemical energy gained from hydrolyzing a single adenosine triphosphate (ATP) molecule for linear motion^13^. Due to these reasons, there has been a fair amount of work on modeling the motion of molecular motors, with modeling techniques falling into two main camps: collective effects of multiple motors along a single track^14–17^, and the effects of the cargo on single motor dynamics^18–21^.

The bulk of the modeling of molecular motors has been produced on the topic of cooperative transport by groups of motors^14–17^, which has been confirmed experimentally to occur in numerous *in vivo* systems^22,23^. Efforts to model cooperative transport involving direct motor-motor interactions so far have been either based on the strongly simplified ASEP (asymmetric simple exclusion process) approach, which typically use constant, load-independent jump rates^16,17^, or are focused on emergent effects stemming from variable motor numbers via detachment and attachment of motors on the motor track^24,25^.

For the effects of single-motor dynamics on cargo dynamics, much research has been focused on stochastic motor hopping models, where the motor makes discrete jumps along a track with thermodynamic constraints on that motion^18–21^. The main constraint is one of *local detailed balance*, which strictly defines the *ratio* of the forward and backward jumping rates. However, this constraint is insufficient to generate a unique rate formulation, forcing a choice. We identify three of the popular model choices in the literature, which, absent a common nomenclature, we label: Glauber^26,27^, Product Asymmetric-Exchange (P-AsEx)^28–31^, and Difference Asymmetric-Exchange (D-AsEx)^18–20^.

In this work we explore cargo transport properties within these models under controlled constraints to illuminate the different consequences of model choice for both single and multiple motor driven transport. Single-motor force-velocity relationships are first examined to demonstrate the varying response of the different motor models under load. We then focus on motor-number scaling behavior of the models near physical parameters which are both experimentally accessible, and still provide a strong response to parameter perturbations. We find that in the special case of a two-motor system that evolves with the D-AsEx rate formulation, the results allow for the motor team to move faster than either motor alone, and so we pay special attention to understand the underlying physics driving the unique behavior. In other cases, many differences are of more easily probed nature, and could be observed directly using existing experimental techniques. We attempt to underscore these differences and propose potential experiments to explore the behavior of analogous systems to determine which model most accurately describes motor-cargo transport in general.

## Methods

**Figure 1:**
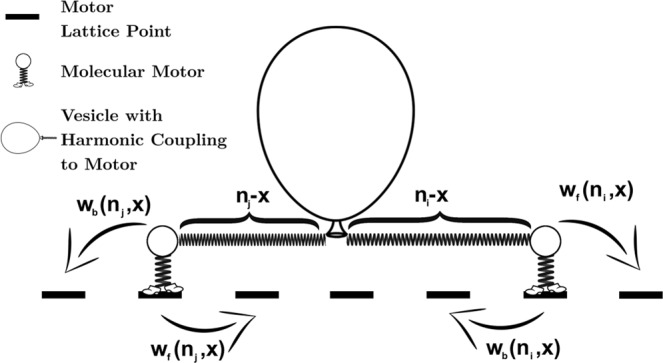
Sketch of modeled one-dimensional motor-cargo system with harmonic coupling showing a single motor moving on a discrete lattice of positions *n* and the cargo moving continuously along a trajectory *x*(*t*).

Cargo transport by motor proteins takes place in a strongly viscous environment where the cargo’s inertia is often negligible. For example, in the extreme case, a large spherical cargo vesicle of radius *R* = 1 *μ*m with transport velocity of *v* ≈ 1 *μ*m s^−1^ in aqueous solution^32^ only has a Reynolds number of Re ≈ 10^−6^. Accounting for the fact that peak velocities of the motor resulting from its stepwise motion may be two orders of magnitude larger^33^ we still remain in a clearly overdamped regime at Re ≤ 10^−4^. For *in vitro* systems in simple aqueous solutions, Brownian dynamics are therefore clearly an appropriate description for the mechanics of this system, but *in vivo* systems often show more complicated viscoelastic behavior due to the crowded environment of the cell^34–38^. While viscoelastic effects are out of the scope of this work, we refer the reader to prior modeling studies on this topic^39,40^. For *in vitro* systems, or as a first approximation to *in vivo* systems, we describe the dynamics of the one-dimensional cargo position *x*(*t*) using Brownian dynamics

1a $$m\ddot{x}=0=F({\bf{n}},x)-{F}_{L}-\gamma \dot{x}+\xi (t)$$

1b $$\langle \xi (t)\rangle =0$$

1c $$\langle \xi (t)\xi (t^{\prime} )\rangle =\frac{2\gamma }{\beta }\delta (t-t^{\prime} ),$$

where *F*(**n**, *x*) is the systemic force on the cargo due to the motors located at sites **n** and cargo at position *x*, *F*_*L*_ is a constant external force, *γ* is the cargo’s drag coefficient, the stochastic force *ξ*(*t*) describes Brownian motion at a thermal energy of 1/*β* = *k*_*B*_*T*, and the cargo mass *m* is taken to zero as inertial forces are considerably smaller than the systemic and drag forces in the overdamped limit. In this formulation, only the particle’s size affects the dynamics of transport via the Stokesian damping constant *γ*, which is proportional to the product of *R* and the dynamic viscosity of the environment. The driving force *F*(**n**, *x*) results from the coupling of the cargo to the motor heads at positions **n** representing on the microtubule via each motor’s linkage. Here, **n**, is a vector of dimensionless motor positions along the microtubule and contains the number of motors, *N*_motors_, elements, as illustrated in Fig. 1. We model the stretching of this linkage upon motor stepping with a harmonic spring force of coupling constant *k*, whose typical stiffness is on the order of 10^−1^ pN/nm^28,41,42^, or in the dimensionless microscopic units from Table 1, roughly 1–10.

**Table 1:** Our system of units used throughout this paper.

Base			Derived		
Length	Energy	Time	*k*	*γ*	*F*
*λ*	*k*_*B*_*T*	*τ*_0_	$\frac{{k}_{B}T}{{\lambda }^{2}}$	$\frac{{k}_{B}T{\tau }_{0}}{{\lambda }^{2}}$	$\frac{{k}_{B}T}{\lambda }$
8 nm	4.3 · 10^−21^ J	1 s	$0.067\frac{{\rm{pN}}}{{\rm{nm}}}$	$0.067\frac{{\rm{pN}}\cdot {\rm{s}}}{{\rm{nm}}}$	0.52 pN

For motor dynamics, we follow prior work with a basic outline given here^18–21^. All derivations and equations from here on will use the dimensionless units defined in Table 1. We first assume that motors attached to a static cargo will adopt a position probability distribution *p*(*n*_*i*_, *x*) following Maxwell-Boltzmann statistics in the long-term, i.e.

2 $$p({n}_{i},x)\propto {e}^{-E({n}_{i},x)},$$

with *E*(*n*, *x*) being the dimensionless energy. Given that the system is thermalized on time scales that are shorter than the jump frequency, the choice to use the Maxwell-Boltzmann distribution is natural^18,20,43^. With the additional constraint that the forward and backward jump rates *w*_f_ and *w*_b_ fulfill detailed balance, we get the following central condition for our rate formulations:

3 $$\frac{{w}_{{\rm{f}}}({n}_{i}-1,x)}{{w}_{{\rm{b}}}({n}_{i},x)}=\frac{p({n}_{i},x)}{p({n}_{i}-1,x)}={e}^{-[E({n}_{i},x)-E({n}_{i}-1,x)]}.$$

The state energy *E*(*n*, *x*) can be defined iteratively by the chemical energy Δ*μ* > 0 gained through ATP hydrolysis and the stretching corresponding to a step of length *λ* by the motor

$$E({n}_{i},x)-E({n}_{i}-1,x)=k({n}_{i}-x-\frac{1}{2})-\Delta \mu .$$

4 $$\begin{array}{rcl}E({n}_{i},x)-E({n}_{i}-1,x) & = & \frac{k}{2}{({n}_{i}-x-1)}^{2}-\frac{k}{2}{({n}_{i}-x)}^{2}-\Delta \mu \\  & = & k({n}_{i}-x-\frac{1}{2})-\Delta \mu .\end{array}$$

We note that, due to the cancellation of the quadratic terms, the difference takes on a linear form. This, combined with the dimensionless units leads to an unintuitive expression for energy. When returning to dimensional units, all lengths will carry with them a step size *λ* in the initial quadratic term and forward, and therefore the dimensional energy will recover the correct units $k\cdot \frac{{k}_{B}T}{{\lambda }^{2}}\cdot {\lambda }^{2}={k}_{B}T$.

### Simulation

Now that we have dynamical evolution equations for both the motor and the cargo, we can use an iterative hybrid technique to calculate the average cargo velocity by alternating between a kinetic Monte Carlo step for motor motion, and a Brownian dynamics step for the cargo motion.

For each timestep, the kinetic Monte Carlo routine is executed as follows. We first calculate the rates for each motor *i* at position *n*_*i*_ and cargo position *x*, to move either forward *w*_*f*,*i*_(*n*_*i*_, *x*) or backward *w*_*b*,*i*_(*n*_*i*_, *x*) using the relevant formulation described below. The probability for *any* motor to move during the timestep *δt* is then calculated as

5 $${P}_{{\rm{hop}}}\approx \delta t\,{w}_{{\rm{tot}}}=\delta t\mathop{\sum }\limits_{i}^{N}\,[{w}_{f,i}({n}_{i},x)+{w}_{b,i}({n}_{i},x)],$$

where $\delta t=\,{\rm{\min }}(\frac{{10}^{-3}}{{w}_{{\rm{tot}}}},\frac{\tau }{10})$, $\tau =\frac{\gamma }{Nk}$ is the cargo relaxation timescale, and *N* is the number of motors attached to the cargo. This constraint ensures both that multiple motor events do not occur during a given timestep, and that the cargo does not appreciably move during this timestep. For simulations where motor heads cannot occupy the same lattice site (SIapp:interacting-motors), the appropriate rates are set to zero if the motor move would result in an overlap of the heads of two adjacent motors. If an event occurs during this timestep, ${\mathscr{U}} < \delta t\,{w}_{{\rm{tot}}}$, where ${\mathscr{U}}$ is a uniform number on the interval [0, 1), then a single motor move is executed randomly with a weighted probabil_*i*_ty *P*_*i*,*j*_ _=_ *w*_*i*,*j*_/*w*_tot_, where *i* represents the individual motor, and *j* represents the direction of hopping.

After completion of the kinetic Monte Carlo portion of the iteration, the cargo then undergoes a simple 1 dimensional Brownian dynamics step,

6 $${x}_{t+\delta t}={x}_{t}+\frac{k\delta t}{\gamma }\mathop{\sum }\limits_{i}^{N}\,({n}_{i}-{x}_{t})-\frac{{F}_{L}\delta t}{\gamma }+\sqrt{2{k}_{B}T\,\delta t/\gamma }{\mathscr{N}},$$

where ${\mathscr{N}}$ is a normally distributed random number with zero mean and a variance of one.

**Figure 2:**
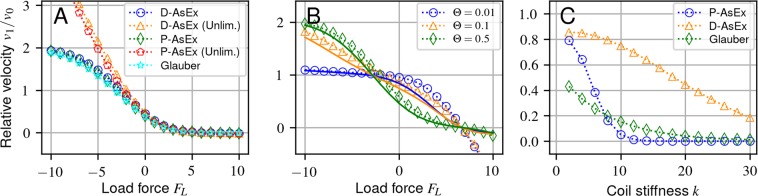
Transport velocities of a cargo pulled by a single motor. System parameters are chosen to provide an unloaded motor velocity of *v*_0_ = 1 *μ*m s^−1^. Default simulation parameters in all three plots in our dimensionless units are: *w*_0_ = 0.154, *γ* = 1.41 × 10^−2^, *k* = 3, and Δ*μ* = 6.7. Error bars are omitted as they lie entirely within the markers. (**A**) Force-velocity curve for each rate formulation generated by subtracting a given load force from the force term *F*(**n**, *x*) in Eq. (1a) acting on the cargo. This convention means that a positive load force *opposes* the forward motion of the motor. Θ^D^ = 0.76, Θ^P^ = 0.71 chosen to ensure equal transport velocities at zero load force. (**B**) Change of force-velocity curve shape from convex to concave depending on Θ demonstrated for D-AsEx (dotted) and P-AsEx (solid) rates. (**C**) Transport velocities for each rate formulation using identical parameters and varying the coiled coil stiffness *k* with Θ = 0.1 in the three models. Values in the *k* → 0 limit are omitted since the cargo velocity will discontinuously go to zero.

However, for the kinetic Monte Carlo step, there are a number of different formulations for the rates *w*_*f*_ and *w*_*b*_ which obey detailed balance, each with different dynamics depending on the input parameters (Fig. 2A–C). We outline three popular rate formulations here, with alterations where necessary.

### Rate formulations

#### Glauber model

The Glauber rates follow an ansatz which strongly resembles the Fermi-Dirac distribution and also fulfills the detailed balance condition of Eq. 3^26,27^.

7a $${w}_{{\rm{f}}}^{{\rm{G}}}({n}_{i},x)=\frac{2{w}_{0}{e}^{\Delta \mu }}{1+{e}^{k({n}_{i}-x+\frac{1}{2})}}$$

7b $${w}_{{\rm{b}}}^{{\rm{G}}}({n}_{i},x)=\frac{2{w}_{0}}{1+{e}^{-k({n}_{i}-x-\frac{1}{2})}}.$$

While not extensively studied in the literature, the Glauber rates have an advantage their simplicity: they do not introduce an additional parameter into the rate formulation.

A more common alternative to the Glauber ansatz is one with a formulation reminiscent of the Arrhenius equation for chemical reaction rates^18–20,28–31^. In this work, we classify this family of reaction rates as asymmetric exponential (AsEx) rates and further consider two variations of this ansatz. In both of the AsEx rate formulations considered here, there is a free parameter $\Theta \in {\mathbb{R}}\cap [0,1]$ which functions as a weighting factor for the relative impact of forces acting via the coiled coil on the forwards versus the backwards rates. In contrast to the previously studied models, however, we present these models in an amended form which removes the unbounded motor velocities under load (Fig. 2A). This amendment is necessary to agree with the finding that motor velocities saturate under forward load^44^. We have therefore adjusted the forward and backward rates to never exceed some maximum rate *αw*_0_*e*^Δ*μ*^, where $\alpha \in {\mathbb{R}}\cap \mathrm{[0,}\,\infty ]$, by introducing Heaviside step functions ${\mathcal H}$ into the rate formulations in a way that maintains the detailed balance constraint. In this work, we will however focus only on the *α* = 2 case for direct comparison to the Glauber rates. We distinguish two variations below, letting *δx* = *n* − *x* represent the displacement between the motor and the cargo.

#### Product Asymmetric Exchange (P-AsEx) model

Here, the parameter Θ splits the change in harmonic potential energy $V(\delta x)=\frac{1}{2}k\,\delta {x}^{2}$, corresponding to a forward or backward jump respectively by factors of Θ and (1 − Θ)^28–31^. Letting the change in effective potential for a jump from site *n* → *n* + 1 be denoted as $\Delta {V}_{P}(\delta x,\Theta )$ $=\frac{k\Theta }{2}({(\delta x+1)}^{2}-\delta {x}^{2})=k\Theta (\delta x+\frac{1}{2})$, the rates can be written

8a $$\begin{array}{rcl}{w}_{f}^{{\rm{P}}} & = & {w}_{0}\exp [\Delta \mu -\Delta {V}_{P}(\delta x,\Theta )]\\  &  & \times \,\exp [ {\mathcal H} (\,-\,\Delta {V}_{P}(\delta x,\Theta )-\,\mathrm{ln}\,\alpha )\cdot (\Delta {V}_{P}(\delta x,\Theta )+\,\mathrm{ln}\,\alpha )]\\  &  & \times \,\exp [ {\mathcal H} (\,-\,\Delta {V}_{P}(\delta x+1,\Theta -1)-\,\mathrm{ln}\,\alpha -\Delta \mu )\cdot (\Delta {V}_{P}(\delta x+1,\Theta -1)+\,\mathrm{ln}\,\alpha +\Delta \mu )]\end{array}$$

8b $$\begin{array}{rcl}{w}_{b}^{{\rm{P}}} & = & {w}_{0}\exp [\,-\,\Delta {V}_{P}(\delta x-1,\Theta -1)]\\  &  & \times \,\exp [ {\mathcal H} (\,-\,\Delta {V}_{P}(\delta x-1,\Theta )-\,\mathrm{ln}\,\alpha )\cdot (\Delta {V}_{P}(\delta x-1,\Theta )+\,\mathrm{ln}\,\alpha )]\\  &  & \times \,\exp [ {\mathcal H} (\,-\,\Delta {V}_{P}(\delta x,\Theta -1)-\,\mathrm{ln}\,\alpha -\Delta \mu )\cdot (\Delta {V}_{P}(\delta x,\Theta -1)+\,\mathrm{ln}\,\alpha +\Delta \mu )],\end{array}$$

where in each expression the first term represents the unlimited rate from prior work, the second limits the forward rates to *w*_0_*αe*^Δ*μ*^, and the third limits the backward rates to *w*_0_*αe*^Δ*μ*^. The existence of a forward (backward) limiting term in the backward (forward) rate is required to maintain detailed balance.

#### Displacement Asymmetric Exchange (D-AsEx) model

Here, Θ splits the step into substeps determining the forward and backward rates respectively of size Θ and (1 − Θ). Letting the effective potential $$\Delta {V}_{D}(\delta x,\Theta )=\frac{k}{2}({(\delta x+\Theta )}^{2}$$$$-\delta {x}^{2})=k\Theta (\delta x+\frac{\Theta }{2})$$, the limited rates are then

9a $$\begin{array}{rcl}{w}_{f}^{{\rm{D}}} & = & {w}_{0}\exp [\Delta \mu -\Delta {V}_{D}(\delta x,\Theta )]\\  &  & \times \,\exp [ {\mathcal H} (-\Delta {V}_{D}(\delta x,\Theta )-\,\mathrm{ln}\,\alpha )\cdot (\Delta {V}_{D}(\delta x,\Theta )+\,\mathrm{ln}\,\alpha )]\\  &  & \times \,\exp [ {\mathcal H} (-\Delta {V}_{D}(\delta x+1,\Theta -1)-\,\mathrm{ln}\,\alpha -\Delta \mu )\cdot (\Delta {V}_{D}(\delta x+1,\Theta -1)+\,\mathrm{ln}\,\alpha +\Delta \mu )]\end{array}$$

9b $$\begin{array}{rcl}{w}_{b}^{{\rm{D}}} & = & {w}_{0}\exp [-\,\Delta {V}_{D}(\delta x,\Theta -1)]\\  &  & \times \,\exp [ {\mathcal H} (-\,\Delta {V}_{D}(\delta x-1,\Theta )-\,\mathrm{ln}\,\alpha )\cdot (\Delta {V}_{D}(\delta x-1,\Theta )+\,\mathrm{ln}\,\alpha )]\\  &  & \times \,\exp [ {\mathcal H} (-\,\Delta {V}_{D}(\delta x,\Theta -1)-\,\mathrm{ln}\,\alpha -\Delta \mu )\cdot (\Delta {V}_{D}(\delta x,\Theta -1)+\,\mathrm{ln}\,\alpha +\varDelta \mu )],\end{array}$$

where, as with the P-AsEx model, the first term represents the unlimited rate, the second limits the forward rates, and the third limits the backward rates. Note that while the rates are nearly identical to the P-AsEx model with a change in the Δ*V* term, the backward rate in the D-AsEx model has a slight change in form compared to that of the P-AsEx model to maintain the correct behavior.

The split employed in the D-AsEx rates can be interpreted to imply Brownian Ratchet dynamics in an asymmetric potential between motor lattice points. This specific rate formulation has been used in particularly by Zimmermann & Seifert^18–20^, and can be considered an attempt to unify the discrete stochastic model of motor dynamics with the fundamental idea of a continuum ratchet model^45^.

We also note that the leading term in the D-AsEx and P-AsEx model can be mapped to between one another via a change in the effective rate constant ${w}_{0}^{{\rm{P}}}={w}_{0}^{{\rm{D}}}{e}^{-\frac{k\Theta }{2}\mathrm{(1}-\Theta )}$. Since $k \sim 3$ in a physical system, this correction is typically quite small (at most ${w}_{0}^{P}/{w}_{0}^{D}={e}^{-\frac{k}{8}} \sim 0.7$). We therefore focus mostly on the D-AsEx model in this paper, with most of the corresponding figures for the P-AsEx model given in the supplement.

## Results

### Single motor

Despite sharing the same detailed balance condition, there are considerable qualitative differences between the models, even for a single motor, depending on the parameter choices. One of the most common ways to experimentally examine motor dynamics is through experiments where a load force is supplied to the cargo via an optical trap, which is typically coupled to a feedback mechanism to maintain constant force. This technique allows for determination of the load on a motor with its average velocity, with these force-velocity curves varying in shape from roughly linear to convex^32,46–51^. We therefore first examine the cargo velocity under a constant load force to directly compare the models in Fig. 2A,B, normalizing by the unloaded motor velocity

10 $${v}_{0}={w}_{0}\cdot ({e}^{\Delta \mu }-1)$$

for ease of comparison between the different models. For ease of comparison in Fig. 2, we have truncated the plots at high load force *F*_*L*_ where the velocity will become negative in the AsEx models.

We choose parameters such that are typical for a processive kinesin motor in a biological system $k=3 \sim 0.2\,pN/nm$^42^, and *γ* for a 1 *μm* diameter vesicle in a fluid with 100x the viscosity of water. The parameters *w*_0_, Δ*μ*, *α*, and Θ, however, require special care since they are not directly measured. We chose Δ*μ* = 6.7 so that the ratio of forward to backward rates of an unloaded motor is roughly 800^48^, which to maintain a typical motor velocity of 1*μm*/*s*, requires *w*_0_ = 0.154. This value for the chemical potential is low compared to what is expected for the energy gained through ATP hydrolysis, though we emphasize that a strict interpretation of *μ* as the change in free energy during hydrolysis leads to nonsensical results such as the velocity being unbounded with increase in relative ATP concentration. The underlying motion of the motor should, at the microscopic level, obey Michaelis-Menten (MM) kinetics, where ATP uptake and consumption are blocked for some duration during a motor head’s stepping event and indeed numerous experiments have demonstrated the MM behavior of molecular motors^32,41,52–55^. However, since these models assume an instantaneous step, a direct recovery of MM is impossible. Instead, this rate limiting must be handled by an appropriate combination of *w*_0_ and Δ*μ*, rather than the more phenomenological MM. We leave Θ as an open parameter as it determines the shape of the force-velocity curve. We set *α* = 2 to make comparison to the Glauber model more direct. We note that this is high compared to experimental values ($\alpha  \sim 1.3$)^48^, but since the motors are rarely under significant forward or backward load in this work, the difference in results are negligible.

In Fig. 2A we use the parameter Θ to equalize the transport velocities for all three rates at zero external force and otherwise identical system parameters. All of the rate formulations show nearly identical force-velocity relationships when the force is positive, i.e. the forces are applied against the motor’s preferred direction of motion. For forces in the motor’s preferred walking direction, we find that the velocities under the Glauber rates and our modified AsEx rates all saturate at a maximum velocity, as we would expect in a real system, whereas the original unlimited AsEx forward rates are inherently unbounded (Fig. 2A).

Despite the nearly identical behavior shown between the models when fixing Θ to high values as in Fig. 2A, the AsEx models exhibit considerably different behavior when the load-splitting parameter Θ is allowed to vary. In both of the AsEx models, varying Θ can dramatically change the inflection and slope of the force-velocity curve near the zero-force limit (Fig. 2B). We also see from Fig. 2B the near equivalence of the two AsEx models at low *k*, when normalized by the unloaded velocity *v*_*o*_. Similar inflection and slope differences have been observed in prior experiments, potentially being driven by an underlying AsEx model^32,46–48,54^. These shape changes can be easily understood by looking at the Θ dependence in the AsEx models with *w*_*f*_ ∝ *e*^−*k*Θ*δx*^: a small Θ leads to a low sensitivity of the forward rate in motor extension, leading to a small response to load force.

Within the models presented, the stall force necessarily evaluates to *F*_stall_ = Δ*μ*, independently of other motor parameters. The stall force is therefore ${F}_{{\rm{stall}}}=6.7 \sim 3.5\,{\rm{p}}N$ for all models, which is at the low end for typical kinesins, but within reported ranges^32,41,48,50,51,56^, though roughly half that reported for the work for which our estimate of Δ*μ* is based^48^. This could indicate that the force, rather than the energy difference, dictates the jumping probabilities, though examination of this hypothesis is beyond the scope of this work.

The models can also exhibit different qualitative behavior under other parameter changes. For example, by varying the coil stiffness *k*, as shown in Fig. 2C, cargo transport velocities can vary considerably between the three rate formulations for identical parameters. While the D-AsEx model has low sensitivity on the coil stiffness for this choice of parameters, the P-AsEx and Glauber models result in an order of magnitude decrease in the cargo velocity and a completely different functional form (Fig. 2C).

It is clear that the models exhibit distinct changes in response under load. In principle, the response to load with known *w*_0_, Δ*μ*, *k*, *γ*, and saturation velocity *w*_0_*e*^Δ*μ*^*α* should be sufficient to uniquely identify if the Glauber or an AsEx model is appropriate. Due to the difficulty of adjusting the coil stiffness of the motor protein *k*, it is potentially difficult to distinguish between the two AsEx models in biological systems, since the differences at biological *k* are small (Fig. 2). Therefore, multiple-motor experiments may provide a more reliable way to experimentally determine the underlying motor mechanics over single-motor force-velocity measurements, as well as illuminating how motors can cooperate under various constraints.

### Two motors

**Figure 3:**
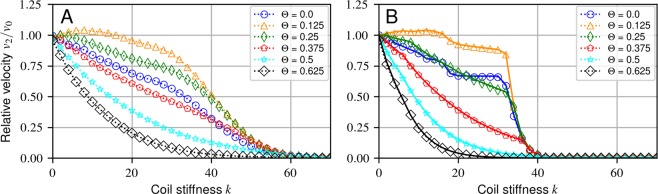
Relative velocities *v*_2_/*v*_0_ for two non-excluding motors and with parameters in our dimensionless units *γ* = 10^−3^, Δ*μ* = 15.0, and *w*_0_ = 4 × 10^−5^. Values are chosen to give an unloaded motor velocity of 1 *μ*m s^−1^. Error bars are omitted as they lie entirely within the markers. (**A**) D-AsEx rates simulation for two non-interacting motors. (**B**) D-AsEx rates simulation (dotted line) vs analytic approximation (full line) with no thermal motion of the cargo. Apparent step-like behavior for small Θ is due to the corresponding step-like behavior in the detailed balance extensions of the model.

When examining cooperativity of motors operating in teams, we discovered interesting behavior in systems of two motors in certain limits: two non-interacting *loaded* motors can move faster (velocity *v*_2_) than each individual *unloaded* motor can alone (velocity *v*_0_) in the D-AsEx model when motors are allowed to occupy the same lattice site, with the effect being most prominent for relatively large values of the motor coil stiffness *k* (Fig. 3A). This effect appears in the low drag ($\gamma \ll 1$), and high biasing (Δ*μ* ~ 10–20) limit, with the effect being most prominent at large coil stiffness (*k* ~ 10–20). This regime might difficult explore in biological systems, due to the need to control the motor intrinsic coil stiffness *k*, potentially by direct mutations or interactions with other molecules or proteins. However, the findings here could be readily useful for engineered motors, where the coil stiffness could be exploited directly to build more efficient teams of motors. The effect also provides a useful way to examine how multiple motors cooperate with the dynamical formulations explored in this work.

To help understand this phenomenon, we take a simpler case of the same system, with the small modification of removing the thermal motion of the cargo (Fig. 3B). By neglecting thermal fluctuations and taking the limit *γ* → 0, the free variable of the cargo position *x* is removed, since the cargo will quickly relax to the equilibrium position between the motors before their next jump, greatly simplifying analysis. This approximation isolates the consequences of coupling of motors from other components of the system in a way that is easier to interpret the observed velocity. Obviously, the coupling is the dominant contribution and there is a clear resulting similarity between 3A and 3B. Naturally, in true physical conditions thermal fluctuations become *more* prominant when reducing the drag, but this does not diminish the effect of the driving. Additionally, the effects of medium viscoelasticity, if included, can not be trivially removed in this manner, as the elasticity introduces another timescale into the system that must be carefully handled. We these caveats considered, now the state of the system can be completely described by the discrete distance between the two motors *ξ*.

By assuming a steady state distribution of the two motors and using the detailed balance constraint, we derived the velocity of the system by calculating the probability *P*_*ξ*_ for the motors to be in state *ξ* with the velocity contribution *v*_*ξ*_, the full details of which are shown in SIapp:two-motor-approximation. Assuming the backward rates are negligible for the most probable configurations (low *ξ*) allows us to solve for the velocities exactly (SIapp:harmonic-potential). The speedup in the D-AsEx model can then be understood by seeing that the probabilities *P*_*ξ*_ decay exponentially in *k*Θ*ξ*^2^, while the velocities grow exponentially in *k*Θ. This allows for some states which, despite their low probability, contribute considerably to the mean velocity due to their exponential growth. An analytic solution also allows us to trivially find the optimal Θ for a given *k* in the D-AsEx model. For physical values $k \sim 1$, Θ ≈ 1/8 maximizes the cooperativity, in good agreement with Fig. 3, though the speedup is marginal at such small *k* with *v*^*D*^/*v*_0_ ≈ 1.008. In general, we find that Θ ≈ 1/8 leads to optimal cooperation between two non-interacting motors in this limit, nearly independently of the *k* value chosen.

We also find that in contrast to the D-AsEx model, the P-AsEx two-motor system monotonically decreases in *k*Θ, where in the unlimited case, $\langle {v}^{P}\rangle \approx {e}^{-\frac{k\varTheta \mathrm{(1}-\varTheta )}{2}}\langle {v}^{D}\rangle$. Essentially, the decrease in the effective rate *w*_0_^*P*^ in the P-AsEx model for a given Θ always counters out the speed gains from the D-AsEx model.

It is important to note that a similar speedup effect has been observed in an analogous system modeling RecBCD enzymes, where two non-interacting motors are directly tethered to one another with a rate splitting that, at first glance, resembles the unlimited P-AsEx model^30,31^. Their speedup is seemingly directly in contrast to our findings here, where the P-AsEx model forbids faster-than-unloaded cooperation. To resolve this apparent contradiction, we note that there are important differences between the work of Stukalin *et al*.^30^ and ours.

The most obvious difference is that the model of Stukalin *et al*. uses a *linear* potential between states, rather than the harmonic potential used in this work. This means all steps have a fixed energy difference, rather than one that grows linearly with increasing motor extension. However, since they consider only states where the motors are adjacent or overlapping, this difference is never realized. More importantly, their rate-splitting formulation is actually fundamentally different than the unlimited P-AsEx splitting in this work, and we find this difference is what drives the disparate behaviors between the two models. While we employ a scheme where the rates always receive the same Θ weighting on the change in energy, their weighting changes depending on if the jump is downhill or uphill in energy, essentially having Θ → 1 − Θ when changing the sign of the energy difference for a jump. When applying our P-AsEx splitting to their model, we find that the velocity similarly monotonically decreases with increasing energy cost, resolving the apparent contradiction. A more rigorous derivation of the mean velocity with a linear potential in the unlimited P-AsEx model shows that 〈*v*/*v*_0_〉 ≈ 2(1 + *e*^*ε*Θ^)^−1^, where *ε* sets the energy scale of a single jump (SIapp:linear-potential).

These model choices culminate in some quantitative and qualitative shifts between our work and theirs. For the parameters we have observed under the D-AsEx model, we can obtaining a maximum of approximate 1.1× speedup, while in their model the effect can go up to 2× for Θ = 0 with increasing energy cost for hopping (effectively *k* in the D-AsEx model). For non-zero Θ, however, both models show an increase in cooperativity at small load-sharing factor up to a certain energy scale, where the motors start becoming anti-cooperative.

### Motor number scaling

Motors often operate in teams much larger than two to move a burdensome cargo. In many cases, the motor heads along the track can significantly hinder one another^57,58^. However, in other cases, the motors’ locations along their tracks are sufficiently far apart to not significantly interact with one-another, such as in intraflagellar transport^59–61^ or motility assays^55,56,62–64^. This limit can also be realized if motors walk along the same microtubule on separate protofilaments. This is mathematically equivalent to each motor being attached to a point which is an integer multiple of the lattice spacing *λ*. We focus here on the non-interacting case, with a simple model of the interacting case detailed in SIapp:interacting-motors.

**Figure 4:**
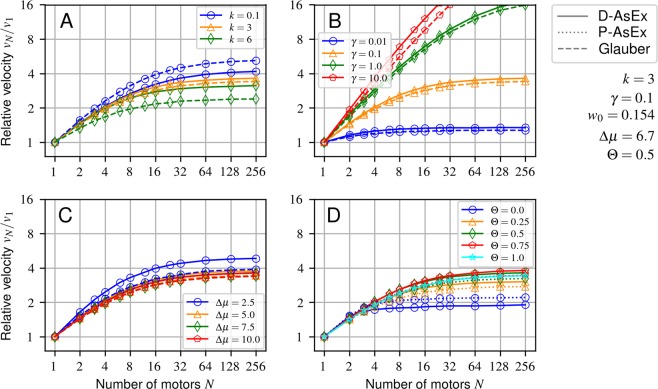
Relative velocities versus number of motors for various model parameters without lattice site exclusion. Parameters are varied in one dimension relative to the central point indicated in the figure. P-AsEx not displayed in A-C to avoid clutter since the plots are nearly identical at small *k*. Error bars are likewise omitted as they lie entirely within the markers. (**A**) Varying motor-cargo coupling *k*, (**B**) cargo damping *γ*. (**C**) ratio of stepping direction via Δ*μ* holding *w*_0_Δ*μ* fixed, (**D**) and load-sharing factor Θ.

When considering the cooperativity of teams of motors, it is useful to measure the velocity of a team of motors normalized by the velocity of a *loaded* single motor *v*_1_. This gives a direct measure of how the velocity scales with the addition of extra motors, with “perfect” scaling being represented by *v*_*N*_/*v*_1_ = *N*. Due to the large parameter space, we do several “scans” about a central point (Fig. 4), varying the motor coil stiffness *k*, jump bias Δ*μ*, load-sharing factor Θ, and the cargo’s viscous drag coefficient *γ* separately. The parameters are chosen to be physically plausible, fit existing experimental data well, and still allow for cooperativity effects to be apparent. For this reason, we keep *k*, Δ*μ*, and *w*_0_ the same as in the single motor comparison (Fig. 2), and additionally select a relatively large Θ = 0.5, since observed motors only respond weakly to loads which exceed the stall force^48,49^. Notably, we choose a drag coefficient (*γ* = 0.1) corresponding to a 1 *μm* diameter vesicle in a fluid with a viscosity roughly 10^3^ times that of water, similar to the viscosity measured in PC12 neurites^65–67^ and blood granulocytes^68^. We choose this seemingly high viscosity as it allows for large cooperativity gains while still being relevant for transport in some biological systems. We also note that increasing the base motor hopping rate *w*_0_ is equivalent to increasing the viscosity when normalized by the single motor velocity–i.e., for a given *w*_0_*γ*, the normalized velocity *v*(*w*_0_*γ*)/*v*_0_ is fixed and so do not include comparisons of *w*_0_ here.

The response of the system to changing *k* is understandably complex, since it is the only parameter to be directly involved in both the motor hopping dynamics and the cargo motion dynamics (Fig. 4A). Increasing *k* leads to a considerable *decrease* in cooperativity with increasing motor number in the Glauber model, but a considerably smaller *decrease* in cooperativity in the D-AsEx model. Notably, the Glauber model does not approach *v*_*N*_/*v*_0_ = 1 for large motor numbers, especially with increasing *k*: ${v}_{N}/{v}_{1} \sim 0.95$ for *k* = 1, but only ~0.3 for *k* = 6. Stiffer motors do incur a cooperativity penalty, similar to the prior findings with mutant kinesins, though without prior observed anti-cooperativity^63,64^. The lack of anti-cooperativity appears to be mainly due to the high drag and intermediate forward rate biasing examined here. Given their use of a standard motility assay, and the reports of a much smaller stall force than reported in most kinesins (3.5 pN), the low drag and low forward rate bias limit might be more appropriate to observe anti-cooperativity. Brief examinations in this limit demonstrate anti-cooperativity with increasing motor stiffness (SIapp:anticooperativity).

The cargo drag coefficient *γ* also has a very strong effect on cooperativity, where increasing the drag increases the cooperativity, as shown in Fig. 4B. This is at first glance possibly counter-intuitive, since *γ* increases the relaxation time and causes the cargo to lag behind the mean motor position for longer. The cargo’s backward position then in-turn lowers the overall average forward jump rate, lowering the mean velocity. However, the velocities are scaled relative to the single-motor velocities, which can also be drastically diminished by increased cargo drag (SIapp:param_scan), counteracting the relaxation time cost. This contrasts the cooperativity observed in the low-drag limit discussed above, where cooperativity is due to the increased hopping rate of the rear motor rather than the increased resistance to motion shown here. The two cooperativity effects happen at different limits of drag where it is unlikely that the two effects ever contribute appreciably in tandem (SIapp:d-asex-gamma). The velocities also saturate at large motor numbers, though the saturation point is strongly parameter dependent. The full cargo relaxation time decreases inversely proportional to the number of motors in the train *γ*/(*N* · *k*), such that, roughly, when $\gamma /(N\cdot k)\ll {e}^{-\Delta \mu }{w}_{0}^{-1}$, the velocity saturates. It is still useful to note that high-drag environments have been useful to study cooperativity since these environments can provide much larger changes in relative velocity over much larger motor numbers. For example, Hunt *et al*.^56^ demonstrate that on motility assays, there is a very clear motor number dependence on transport speed when $\gamma  \sim 0.1$, while Gagliano *et al*.^62^ find a motor number scaling behavior for beads drawn in xantham solution ($\gamma  \sim 0.23$) that is similar to that of the *γ* = 0.1 curve shown here.

The final way to directly adjust a transport timescale, as with *k* and *γ*, is by adjusting the forward jump bias Δ*μ* (here holding *w*_0_Δ*μ* fixed), as shown in Fig. 4C. The cooperativity is almost entirely unchanged between the models, excluding the extreme low biasing (unloaded rate ratio ${e}^{\varDelta \mu }={e}^{2.5} \sim 12$), indicating that even with relatively high loads, backward stepping is too rare to cause a dramatic impact on transport.

It is clear that adjusting the timescales, forces, and energies involved in transport can dramatically effect the velocities of cargo transport. It is also clear from this work that the model choice can be equally, if not more important in deciding the net transport velocity given these known properties of a system, so we further observe the effects of varying the main model parameter Θ on transport. We find that for the chosen parameters, the scaling behavior is almost independent of Θ, with the exception of Θ = 0, which saturates at low motor numbers due to a high single-motor velocity (Fig. 4D). Given the slow saturation with motor number seen in prior high drag experiments^62^, this is additional support that an AsEx model for kinesins should have a relatively high Θ, as also suggested by the force-velocity curves.

## Concluding Remarks

There are clear and distinct differences in the physical behavior of motor-cargo systems depending on the underlying rate formulation chosen. These choices can allow for both unintuitive effects, such as the faster-than-unloaded behavior shown in the two-motor D-AsEx system at low viscosity, drastic qualitative and quantitative shifts in the force-velocity relationship for a single motor under load, and the less drastic changes in cooperativity of large numbers of motors working in teams. However, when constraining the model to roughly match the force-velocity relations given by experiments, these differences narrow, and the model differences become small but not insignificant.

We find that if AsEx models are used, a value of $\Theta  \sim 0.5$ is reasonably appropriate to describe the force-velocity relationships and multiple motor scaling relationships observed in the literature so far for small forces, with the behavior of the stochastic model being driven by primarily the force-velocity relationship. However, given their simplicity, these models can not fully account for the observed force-velocity relationships over the entire range measured experimentally. The issue arrises because Δ*μ* sets both the forward rate bias as well as the stall force, which is actually underestimated. This yields a factor of two difference of the slope of the model’s force-velocity relationship over the relevant experimental range. Future works involving these stochastic models need to address this deficiency to give a more precise quantitative predictions, the qualitative behavior observed in this work is relatively robust and should remain valid.

For discerning the differences between the models, the largest quantitative shift of motor number scaling behavior is in the coil stiffness *k*. While it’s been demonstrated that under low drag circumstances, high stiffness motors become antagonistic, future experiments, as those done by *et al*.^62^, could examine this behavior under higher drag conditions where there is considerable more capacity for cooperativity predicted by these models. Since the models show the clearest differences when adjusting the spring stiffness *k*, measurement of the force-velocity relationship for a motor with artificially increased stiffness and high drag experiments jointly could illuminate the appropriate microscopic model most effectively. Alternatively, for engineered motors, the difficult restraints on Θ and the coil stiffness *k* could be lifted directly, allowing for novel tuning of the cooperativity and force-velocity relationships.

## Supplementary information

Supplementary Information
