# Supplementary material for: The Impact of Rate Formulations on Stochastic Molecular Motor Dynamics
Source: Sci Rep. 2019 Dec 5;9:18373. doi: 10.1038/s41598-019-54344-2 (PMC6895049; doi:10.1038/s41598-019-54344-2)
Supplement: Supplementary file 1 — Supplementary Information [file 41598_2019_54344_MOESM1_ESM.pdf]

# The Impact of Rate Formulations on Stochastic Molecular Motor Dynamics

**R. Blackwell[1], D. Jung[1], M. Bukenberger[1], and A.-S. Smith[1,2,*]**

[1]PULS group, Physics Department and Interdisciplinary Center for Nanostructured Films, Friedrich-Alexander University Erlangen-Nürnberg, Cauerstrasse 3, 91058 Erlangen, Germany
[2]Group for Computational Life Sciences, Division of Physical Chemistry, Insitut Ru ¯der Bošković, Bijenička cesta 54, 10000 Zagreb, Croatia
[*]smith@physik.fau.de

## ABSTRACT

Supplementary information for manuscript.

# 1 Low-viscosity two-motor analytic approximation

We let $P_\xi$ be the unnormalized probability for the two motor heads to be at a distance of $\xi$ from each other, and assume a steady state distribution so that $\frac{\mathrm{d}P_\xi}{\mathrm{d}t} = 0 \,\forall\, \xi$. Starting with some unknown $P_0$ we can iteratively calculate $P_{n+1}$ as a function of all $P_{m\leq n}$ using the detailed balance constraints,

$$P_1 = 2P_0 \frac{w_\mathrm{f}(0) + w_\mathrm{b}(0)}{w_\mathrm{f}(-\frac{1}{2}) + w_\mathrm{b}(\frac{1}{2})} \tag{1a}$$

$$P_2 = P_1 \frac{w_\mathrm{f}\left(\frac{1}{2}\right) + w_\mathrm{f}\left(-\frac{1}{2}\right) + w_\mathrm{b}\left(\frac{1}{2}\right) + w_\mathrm{b}\left(-\frac{1}{2}\right)}{w_\mathrm{f}(-1) + w_\mathrm{b}(1)} - 2P_0 \frac{w_\mathrm{f}(0) + w_\mathrm{b}(0)}{w_\mathrm{f}(-1) + w_\mathrm{b}(1)} \tag{1b}$$

$$P_{\xi+1} = P_\xi \frac{w_\mathrm{f}\left(\frac{\xi}{2}\right) + w_\mathrm{f}\left(-\frac{\xi}{2}\right) + w_\mathrm{b}\left(\frac{\xi}{2}\right) + w_\mathrm{b}\left(-\frac{\xi}{2}\right)}{w_\mathrm{f}\left(-\frac{\xi+1}{2}\right) + w_\mathrm{b}\left(\frac{\xi+1}{2}\right)} - P_{\xi-1} \cdot \frac{w_\mathrm{f}\left(\frac{\xi-1}{2}\right) + w_\mathrm{b}\left(-\frac{\xi-1}{2}\right)}{w_\mathrm{f}\left(-\frac{\xi+1}{2}\right) + w_\mathrm{b}\left(\frac{\xi+1}{2}\right)}, \tag{1c}$$

which can be solved exactly in terms of $P_0$

$$P_{\xi>0} = 2P_0 \prod_{i=0}^{\xi-1} \frac{w_f\left(\frac{i}{2}\right) + w_b\left(-\frac{i}{2}\right)}{w_f\left(-\frac{i+1}{2}\right) + w_b\left(\frac{i+1}{2}\right)}. \tag{2}$$

Similarly, the velocity of the cargo depending on the current motor configuration can be calculated from the jump rates as

$$v_\xi = \frac{1}{2}\left(w_\mathrm{f}\left(\frac{\xi}{2}\right) + w_\mathrm{f}\left(-\frac{\xi}{2}\right) - w_\mathrm{b}\left(\frac{\xi}{2}\right) - w_\mathrm{b}\left(-\frac{\xi}{2}\right)\right), \tag{3}$$

and the long-term velocity under an equilibrium motor configuration is then obtained by summing over all motor configurations weighted by their respective probabilities

$$\langle v \rangle = \frac{\sum_{\xi=0}^{\infty} v_\xi \cdot P_\xi}{\sum_{\xi=0}^{\infty} P_\xi} = \sum_{\xi=0}^{\infty} v_\xi \cdot p_\xi, \tag{4}$$

where $p_\xi$ is the unit normalized probability such that $\sum_{\xi=0}^{\infty} p_\xi = 1$.

# 2 Two-motor velocity with harmonic potential

When considering motors with a high forward jump bias ($\Delta\mu \gg 1$) and vanishing drag in the unlimited variants of the AsEx models, the velocity can be solved exactly. In both of the AsEx models, setting the $w_b$ terms to zero and using the derivation outlined above, it is trivial to show that the normalized probabilities

$$p_\xi = \frac{(2-\delta_{\xi,0})e^{-\frac{k\Theta}{2}\xi^2}}{\sum_{\xi=0}^{\infty}(2-\delta_{\xi,0})e^{-\frac{k\Theta}{2}\xi^2}}, \tag{5}$$

where the sum in the denominator is an elliptic theta special function and can be written $\theta_3(0, e^{-\frac{k\Theta}{2}})$.

The velocity contribution of a given configuration $v_\xi$ in this limit for the P-AsEx model can be simply written

$$v_\xi^\mathrm{P} = w_0 e^{\Delta\mu} e^{-\frac{k\Theta}{2}} \cosh\left(\frac{k\Theta\xi}{2}\right). \tag{6}$$

Then, putting the velocity and probabilities together, we arrive at the mean velocity in the low $\gamma$ and high $\Delta\mu$ limit

$$\begin{aligned} \langle v^P \rangle &= \frac{w_0 e^{\Delta\mu} e^{-\frac{k\Theta}{2}}}{\theta(0, e^{-\frac{k\Theta}{2}})} \sum_{\xi=0}^{\infty} (2-\delta_{\xi,0}) e^{-\frac{k\Theta}{2}\xi^2} \cosh\left(\frac{k\Theta\xi}{2}\right) \\ &= w_0 e^{\Delta\mu} e^{-\frac{3}{8}k\Theta} \frac{\theta_2(0, e^{-\frac{k\Theta}{2}})}{\theta_3(0, e^{-\frac{k\Theta}{2}})}. \end{aligned} \tag{7}$$

This function monotonically decreases in $k\Theta$, and should act as a maximum velocity for a two-motor system, showing that the P-AsEx model as formulated cannot result in a two-motor speedup relative to the unloaded motors.

The D-AsEx velocity can similarly be calculated using the generalized velocity derivation outlined above. However, since the two unlimited AsEx models can be mapped between one another, we can simply write

$$\left\langle v^D \right\rangle = e^{\frac{k\Theta(1-\Theta)}{2}} \left\langle v^P \right\rangle = w_0 e^{\Delta\mu} e^{\frac{1}{2}k\Theta(\frac{1}{4}-\Theta)} \frac{\theta_2(0, e^{-\frac{k\Theta}{2}})}{\theta_3(0, e^{-\frac{k\Theta}{2}})} \tag{8}$$

Noting that for $k\Theta \lessapprox 4$ the ratio of the elliptic theta functions is $\approx 1$, we can approximate

$$\left\langle v^D \right\rangle \approx w_0 e^{\Delta\mu} e^{\frac{1}{2}k\Theta(\frac{1}{4}-\Theta)} \tag{9}$$

for small $k\Theta$. This clearly allows for speedups for any $\Theta < 1/4$ with the optimal $\Theta = 1/8$.

## 3 Two-motor velocity with linear potential

Since the prior work on modeling the RecBCD protein observed a two-motor speedup used a linear potential between the motor heads, it is useful to explore if this is possible using a linear potential but with a cargo intermediary in the unlimited P-AsEx formulation. The potential can be simply written $V(\delta x) = \varepsilon\,|\delta x|$, where $\varepsilon$ sets the energy scale of a single jump. The forward rate is then $w_f(\delta x) = w_0 e^{\Delta\mu} e^{-\varepsilon\Theta(|\delta x+1|-|\delta x|)}$. Noting that the forward rate can take on one of three values since the displacement can only take integer and half-integer values

$$w_f(\delta x \le -1) = w_0 e^{\Delta\mu} e^{\varepsilon\Theta} \tag{10a}$$

$$w_f(\delta x = -\frac{1}{2}) = w_0 e^{\Delta\mu} \tag{10b}$$

$$w_f(\delta x \ge 0) = w_0 e^{\Delta\mu} e^{-\varepsilon\Theta}, \tag{10c}$$

and setting the backward rates $w_b = 0$, the mean velocity can be simply calculated using the relations from Equations SI2 and SI4

$$\langle v \rangle = w_0 e^{\Delta\mu} \frac{e^{-\varepsilon\Theta}\left(1 + 2\sinh(\varepsilon\Theta)\right)}{1 + \sinh(\varepsilon\Theta)} \approx \frac{2 w_0 e^{\Delta\mu}}{1 + e^{\varepsilon\Theta}}. \tag{11}$$

As with the harmonic potential in the unlimited P-AsEx model, this monotonically decreases in $\varepsilon\Theta$.

## 4 Single motor response in reference set

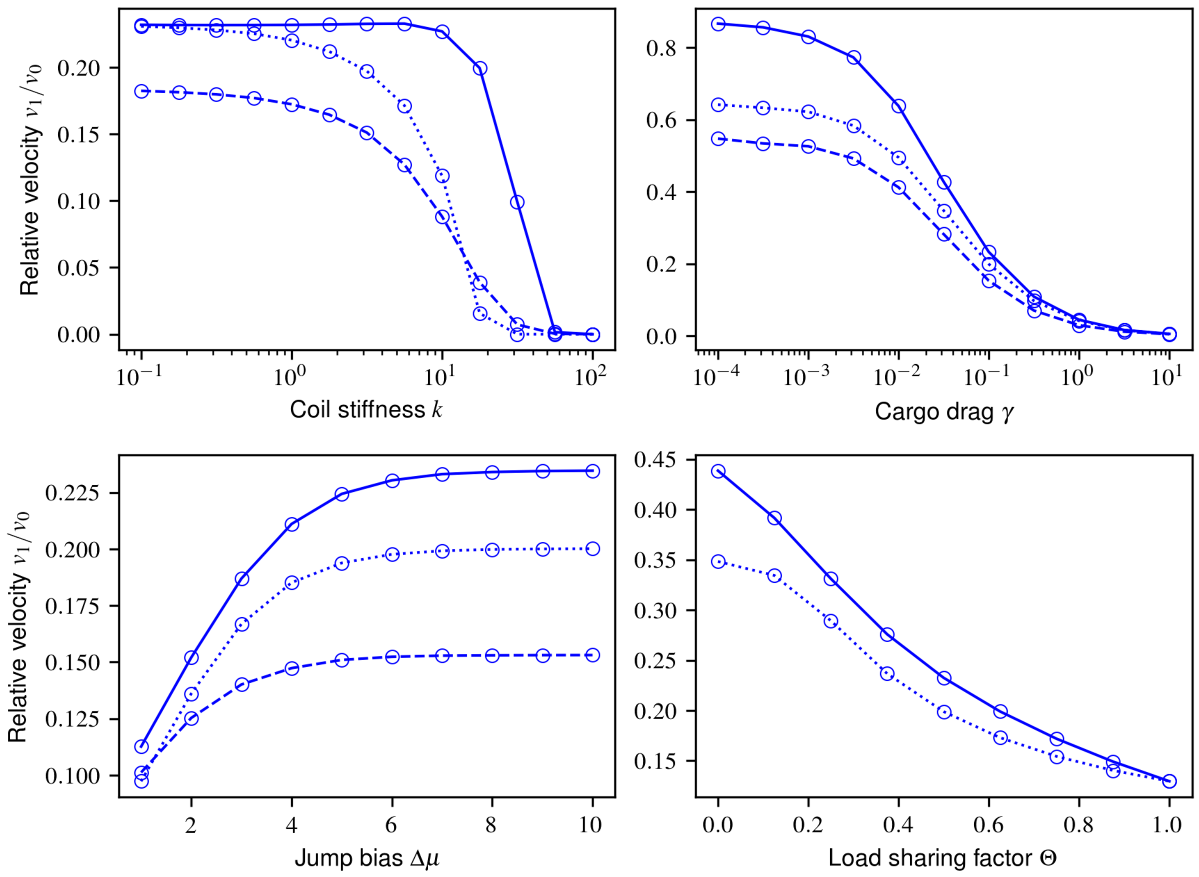


Relative velocities $v_1/v_0$ for single motors about the central point $\gamma = 1$, $\Delta\mu = 15$, $w_0 = 4 \times 10^{-5}$, and $k = 3$. D-AsEx shown in solid lines, P-AsEx in dotted lines, and Glauber in dashed lines. Scan in coil stiffness $k$ (upper left), cargo drag $\gamma$ (upper right), jump bias $\Delta\mu$ (lower left), and load sharing factor $\Theta$ (lower right).

## 5 Two-motor velocities in P-AsEx formulation

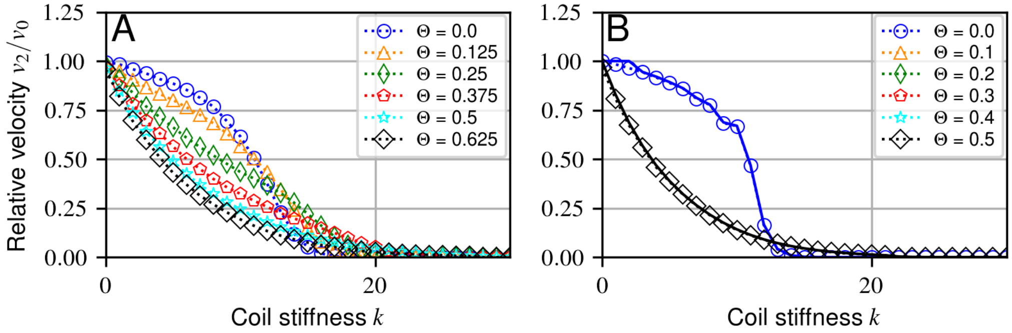


Relative velocities $v_2/v_0$ for two non-excluding motors with parameters in our dimensionless units $\gamma = 10^{-3}$, $\Delta\mu = 15$, and $w_0 = 3.824 \times 10^{-5}$. ***(A)*** P-AsEx rates simulation for two non-interacting motors. ***(B)*** P-AsEx rates simulation (dotted line) vs analytic approximation (full line) with no thermal motion of the cargo.

## 6 Two-motor velocities for varying drag

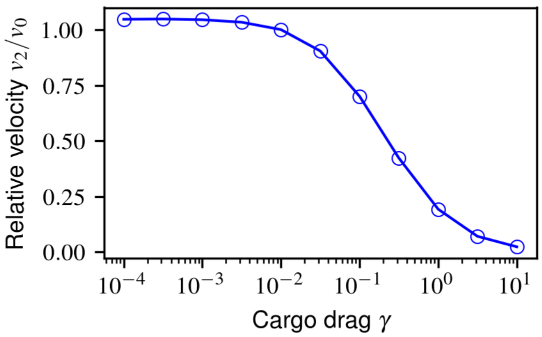


Relative velocities $v_2/v_0$ for two non-excluding motors with parameters in our dimensionless units $k = 10$, $\Delta\mu = 15$, $\alpha = 2$, $\Theta = 0.125$ and $w_0 = 3.824 \times 10^{-5}$.

## 7 Anti-cooperativity

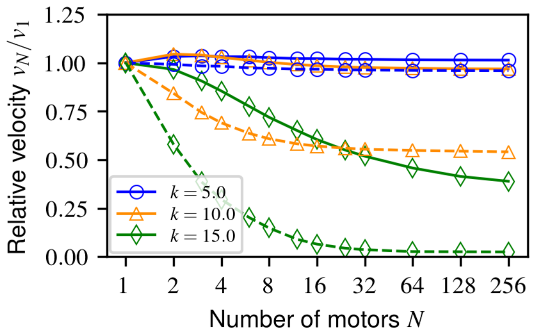


Relative velocities $v_N/v_0$ for two non-excluding motors with parameters in our dimensionless units for varying values of $k$, $\Delta\mu = 4.0$, $w_0 = 2.29$, $\alpha = 2$, $\gamma = 10^{-3}$ and $\Theta = 0.5$. D-AsEx model shown in solid line and Glauber in dashed.

When $k \sim \Delta\mu$ in the low drag limit ($v_1 \sim v_0$), the potential for anti-cooperative behavior between motors arises, even when the motors do not directly interact. In both the D-AsEx and Glauber models, there is clear strong anti-cooperative behavior observed for high stiffness motors $k \sim 6.7$pN nm$^{-1}$.

## 8 Interacting Motors

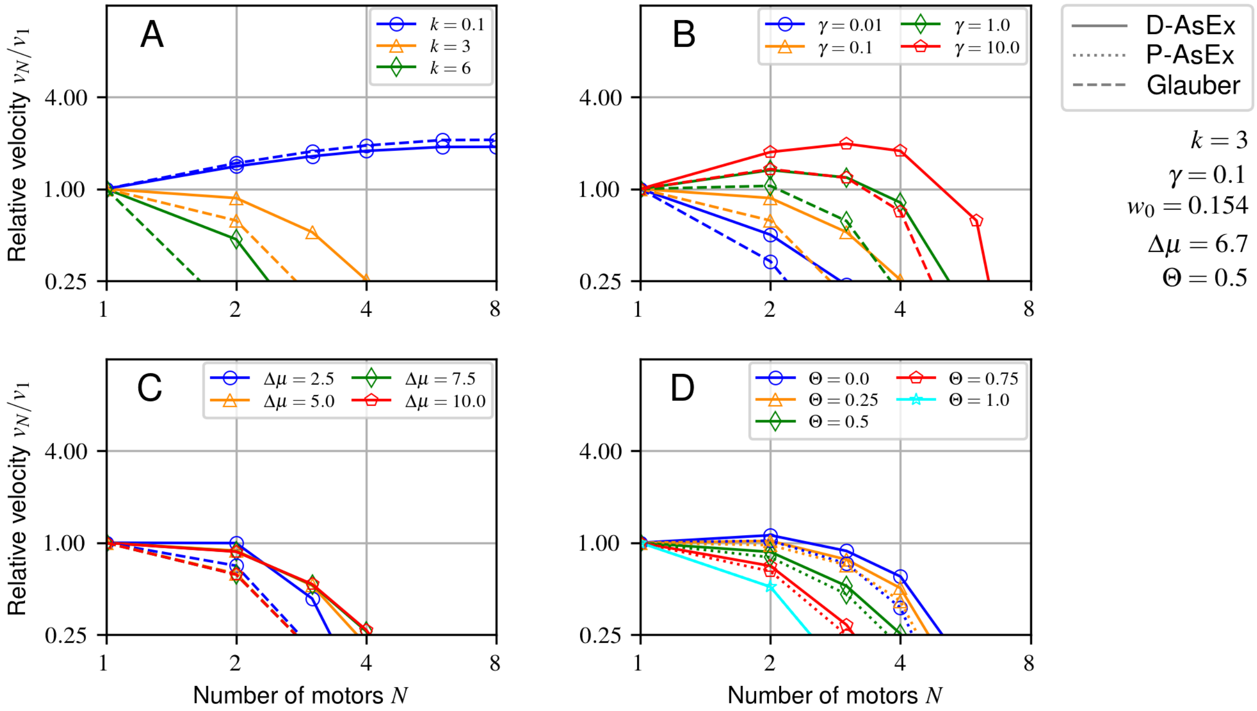


**Figure 1.** Relative velocities versus number of motors for various model parameters with excluded volume. Parameters are varied in one dimension relative to the central point indicated in the figure. P-AsEx not displayed in A-C to avoid clutter since the plots are nearly identical at small $k$. ***(A)*** Varying motor-cargo coupling $k$, ***(B)*** cargo damping $\gamma$, ***(C)*** relative probability of backward stepping via $\Delta\mu$, ***(D)*** and load-sharing factor $\Theta$.

Here, we assume that the motor heads are not allowed to occupy the same lattice position, and that they are all attached at the some point to some cargo. This is an overly simplistic assumption, since multiple motors will not be able to attach to the same site on the cargo, but a full exploration of the effects of this positioning is beyond the scope of this work. This limit is still however useful to show the large differences in model behaviors when motor collisions are included. Due to the relatively high coil stiffness compared to the jump bias, it's perhaps not surprising that cooperativity only arises in the low stiffness (Fig. 1A) or high drag limits (Fig. 1B).
